# Cuprotosis Patterns Are Associated with Tumor Mutation Burden and Immune Landscape in Lung Adenocarcinoma

**DOI:** 10.1155/2022/9772208

**Published:** 2022-11-23

**Authors:** Tingting Liu, Liangliang Cai, Hujia Hua, Xingyu Jiang, Xintian Xu, Tianyi Zhang, Wenqing Huang, Li Qian, Hua Bai, Jianchun Duan

**Affiliations:** ^1^College of Veterinary Medicine, Yangzhou University, Yangzhou 225001, China; ^2^Institute of Translational Medicine, Medical College, Yangzhou University, Yangzhou 225001, China; ^3^Jiangsu Key Laboratory of Experimental and Translational Non-coding RNA Research, Yangzhou, China; ^4^CAMS Key Laboratory of Translational Research on Lung Cancer, State Key Laboratory of Molecular Oncology, Department of Medical Oncology, National Cancer Center/National Clinical Research Center for Cancer/Cancer Hospital, Chinese Academy of Medical Sciences Peking Union Medical College, Beijing 100021, China; ^5^Department of Central Laboratory, Shanghai Children's Hospital, Shanghai Jiaotong University School of Medicine, Shanghai, China

## Abstract

**Background:**

The association involving cuprotosis, molecular subtype, and specific immune cell groups in the tumor microenvironment has been focused on by more recent studies. In lung adenocarcinoma (LUAD), the potential functions of cuprotosis remain elusive.

**Methods:**

The cuprotosis regulations and tumor immune profile of 567 LUAD patients and the correlation between the cuprotosis patterns and the immune landscape were comprehensively evaluated. The cuprotosisScore was calculated using principal component analysis (PCA). The prognostic significance of the cuprotosisScore was evaluated by Cox regression statistics analysis.

**Results:**

Five cuprotosisClusters (named mc1, 2, 3, 4, 5)—characterized by differences in expression of immunomodulatory genes, mRNA, or lncRNA expression, and prognosis were identified. We established cuprotosisScore to quantify the cuprotosis pattern of individual LUAD patients. As is shown in further analyses, the cuprotosisScore was a relatively potential independent prognostic factor of LUAD involved in mc1. Finally, the prognostic value of the cuprotosisScore and its association with tumor immune microenvironment (iTME) of LUAD in five cuprotosisClusters were verified.

**Conclusions:**

We demonstrated the correlation between cuprotosis modification, the molecular subtype, and tumor immune landscape in LUAD. The cuprotosisCluster with high cuprotosisScore and high tumor mutation burden (TMB) was identified with a good prognosis and immune functions. The comprehensive evaluation of cuprotosis patterns in individual LUAD patients enhances the understanding of iTME and gives a new insight toward improved immune treatment strategies for LUAD patients.

## 1. Background

The requirement of copper as a helper for essential enzyme function has been recognized in human cells. However, intracellular copper concentrations are kept very low by active homeostatic mechanisms that work across concentration gradients to prevent the accumulation of free intracellular copper that is detrimental to cells. The mechanisms of copper-induced cytotoxicity had been well explored [1]. A clear picture of the mechanisms underlying copper-induced toxicity emerged by targeting lipoylated TCA cycle proteins: LA pathway (FDX1, LIAS, LIPT1, and DLD) and PDH complex (DLAT, PDHA1, PDHB, MTF1, GLS, and CDKN2A).

Immune checkpoint inhibitor therapy (ICT, mainly PD-1/PD-L1 mono-antibody therapy) is promising in the clinical treatment of lung adenocarcinoma (LUAD) [2, 3]. However, not all LUAD patients show an effective clinical response or even drug resistance to ICT therapy [4]. In many malignant cancer types, a large number of tumors are intrinsic, [2] for example, the effective responses to ICT therapy occur when the TIME is characterized by a high portion of CD8+ T cells and while none occur when there is low CD8+ T cell infiltration [5, 6]. It is important to explore the related characteristics with the TIME or tumor immune microenvironment (iTME) of LUAD that drives the ICT effective clinical response [7, 8] or even the clinical treatment strategies of immune-oncology therapies [9, 10].

In this investigation, we integrated the clinical and molecular data of 461 LUAD cancer patients to comprehensively evaluate the cuprotosis patterns and iTME. Five distinct cuprotosis regulations were identified, and we were surprised to find that they had distinct immune characteristics and prognoses, showing the key roles of cuprotosis in the development of individual iTME in LUAD patients. We then quantified the cuprotosis of individual LUAD cancer patients by evaluating the gene patterns of cuprotosis regulators.

## 2. Methods

### 2.1. Molecular and Clinical Data

From the genomic Data Commons (https://portal gdc cancer gov/.) [11], RNA sequencing data (fpkm and count values) were retrieved for clinical data, genetic mutations, and expression analysis. By consulting an annotation file, the Ensembl gene IDs from the RNA-seq data were changed into the gene symbols (https://www.gencodegenes.Org/human/release22.html). The xena online tool (https://xena ucsc edu/) [12] was used to retrieve the CNV (Copy Number Variation) data. We followed the methods of Zhong et al [13].

### 2.2. Model-Based Clustering Technique for Cuprotosis Regulators

Model-based clustering analysis, performed in the R package/mclust [14], was used to discover cuprotosis modification patterns [15] on the basis of expression of 10 cuprotosis regulators genes. Considering the metric log2 (fpkm +1), gene expression levels were assessed. The Bayesian information criteria were used in this program to calculate the ideal number of clusters.

### 2.3. Gene Set Variation Analysis

For investigating the variations in biological procedures among the cuprotosis modification techniques, GSVA (Gene Set Variation Analysis), an unsupervised and nonparametric technique that is frequently used to estimate pathway differences in samples of expression datasets, was applied. From the Molecular Signatures Database, the c2.cp. kegg. v6.2. symbols gene sets were retrieved (MSigDB) for GSVA. *p* < 0.05 was set for statistical significance.

### 2.4. Determination of Differentially Presented Genes among Cuprotosis Clusters

We grouped 597 patients into cuprotosis clusters on the basis of expression of 10 cuprotosis genes in order to find genes involved in the control of cuprotosis modification. Considering the raw fpkm values from the RNA sequencing data, the R/limma program was considered for identifying the DEGs (differentially expressed genes) in these clusters. Genes with adjusted *p* > 0.05 are referred to as DEGs with around two-fold alterations in the expression.

### 2.5. Formation of Cuprotosis Gene Signature

We used a methodology to calculate each patient's unique cuprotosis alteration technique (cuprotosisScore). Following is how the cuprotosisScore was calculated. In order to evaluate the overlapping DEGs, we first determined the overlying DEGs between cuprotosis clusters and divided LUAD patients into a variety of groups by considering model-based clustering. The cuprotosisScore was determined as follows. We initially retrieved the overlapping DEGs between cuprotosis clusters and used model-based clustering to split the LUAD patients into several groups in order to analyze the overlapping DEGs. At last, the Genomic Grade Index-like methodology was used to define the cuprotosisScore [16–18]:

At last, the Genomic Grade Index was used to define the cuprotosisScore.

(1) $$\begin{array}{c} cuprotosisScore=\sum \left(PC1i+PC2i\right). \end{array}$$

Here, *i* represents the overlapping gene expression having a significant prognosis of DEGs among clusters of cuprotosis.

### 2.6. Correlation between cuprotosisScore and Other Related Biological Procedures

Considering the gene sets presented by Mariathasan et al. [19], Spearman's correlation method was carried out for determining the linkage between cuprotosisScore and other related biological procedures, such as angiogenesis signature, pan-fibroblast transforming growth factor-*β* response signature, Wnt targets, epithelial-mesenchymal transition markers, DNA damage repair, nucleotide excision repair, DNA replication, effector CD8 T-cell signature, mismatch repair, antigen processing machinery (APM), and immune checkpoint.

### 2.7. Statistical Analysis

In order to assess the statistical significance, the Kruskal-Wallis test was considered for three or more groups, and the *χ*2 test was considered to assess any links between categorical variables. Through Spearman's correlation analysis, the correlation coefficient was computed. In order to assess the statistical significance of differences, the Kaplan-Meier technique was considered for building survival curves and the log-rank test was considered. The mutation landscape of the TCGA-LUAD cohort and immunotherapeutic cohort was shown using the oncoplot function of the R package/maftools. *p* > 0.05 significance level was considered for both sides' tests. In every study, the V.4.1.0 (http://www R-project.org.) was considered.

## 3. Results

### 3.1. The Cuprotosis Regulators in LUAD: Molecular Characteristics and Clinical Relevance

**Figure 1:**
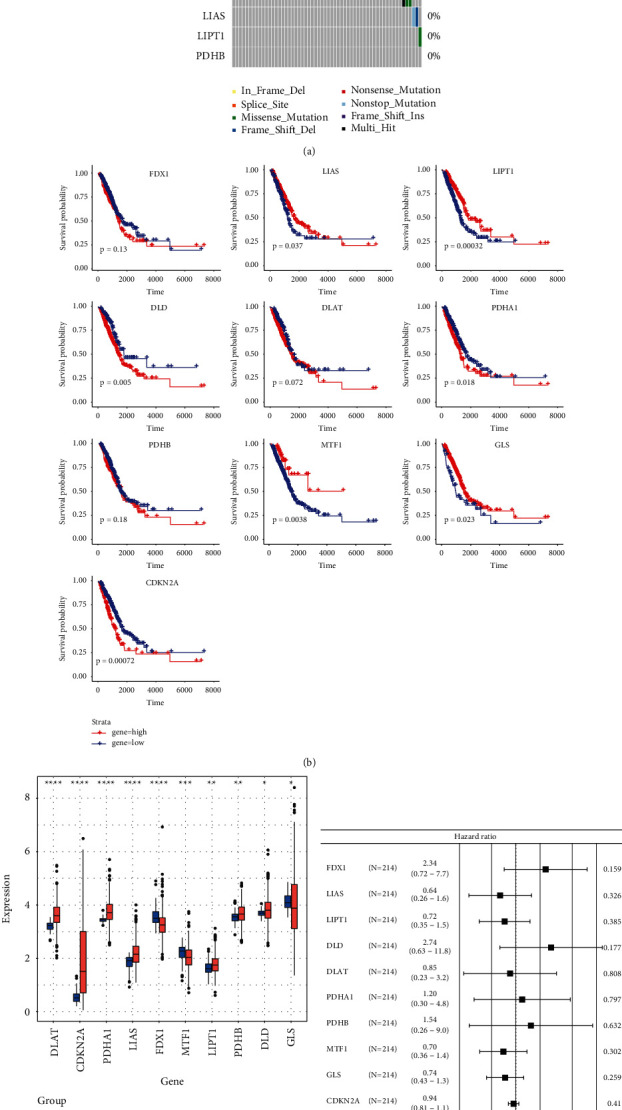
Clinical relevance and molecular characteristics of cuprotosis regulator genes in LUAD. (a) The mutation landscape of 10 cuprotosis regulator genes in TCGA-LUADs; (b) the overall survival of high or low expression of cuprotosis regulators in LUADs; (c) the gene expression alterations among cuprotosis regulators; tumor (normal) was indicated in red (blue). ANOVA test: the asterisks represented the statistical *p* value (^*∗*^*p* < 0.05; ^*∗∗*^*P* < 0.01; ^*∗∗∗*^*P* < 0.001); (d) for clinical relevance evaluation, a Cox model analysis shows positively related genes.

Based on published literature, cuprotosis is regulated by targeting 10 lipoylated TCA cycle proteins: LA pathway (FDX1, LIAS, LIPT1, and DLD) and PDH complex (DLAT, PDHA1, PDHB, MTF1, GLS, and CDKN2A) were highlighted. The frequency of cuprotosis regulator changes in LUAD was investigated using somatic mutations. Only 58 of 567 samples had cuprotosis regulator mutations, indicating that the complete average mutation frequency of cuprotosis regulators was lower (see Figure 1(a)). The survival curve of the 10 cuprotosis regulators was then examined, and it was shown that 8/10 cuprotosis regulators had a substantial influence (*p* < 0.05) on LUAD patients (see Figure 1(b)). The cuprotosis regulators' mRNA expression levels in LUAD and surrounding tissues were also investigated, and it was discovered that 10 of the 10 cuprotosis regulators were differently expressed with *p* < 0.05 (see Figure 1(c)). For clinical relevance evaluation, we execute a Cox model which shows that overall, 10 cuprotosis genes have HR score = 0.6 (see Figure 1(d)). The expressional and genetic differences in cuprotosis regulators were significantly diverse between LUAD and surrounding tissues, indicating that cuprotosis regulator expression imbalance plays a critical role in the formation and progression of LUAD.

### 3.2. The Cuprotosis Modification Patterns Mediated by 24 Cuprotosis Regulators

**Figure 2:**
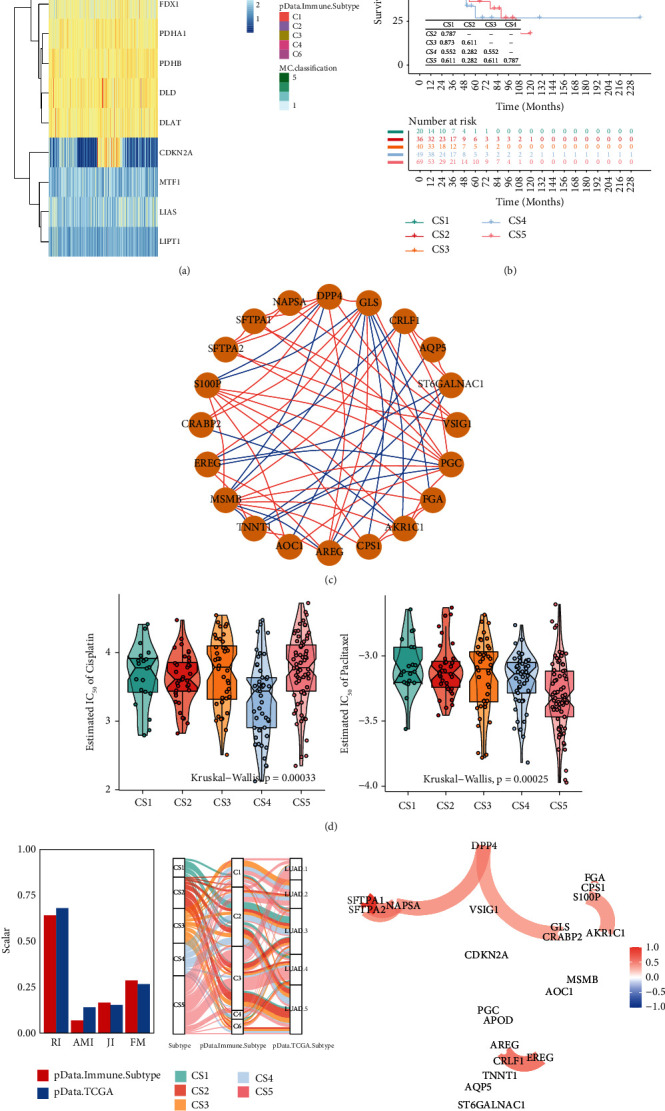
The cuprotosis patterns in LUAD and biological characteristics of cuprotosis subtypes. (a) Model-based clustering of LUAD yields five subtypes in the LUAD dataset. MC1, cluster1; MC2, cluster2; MC3, cluster3; MC4, cluster4; MC5, cluster5; (b) comparison of prognosis among four cuprotosis subtypes (Kaplan-Meier analysis); (c) PPI network based on 23 COX DEGs; (d) Boxviolins for estimated IC50 of Cisplatin and Paclitaxel among 5 identified subtypes of lung cancer; (e) agreement of 5 identified subtypes of lung cancer with classification and pathological stage in LUAD cohort; (f) PPI network based on 23 COX DEG using network.

The 10 cuprotosis regulators' expression was used to categorize LUAD patients using model-based clustering. We found five different RNA methylation modification patterns (called cuprotosis clusters mc1-mc4), with 118 cases in mc1, 129 cases in m6c2, 53 cases in mc3, 53 cases in mc4, and 85 cases in mc5 (see Figure 2(a)). Two risk factors for overall survival (OS) (CDKN2A and GLS) were among the cuprotosis regulators with the largest variations across subtypes. As a result, it is no surprise that mc4 had a poor prognosis (see Figure 2(b)).

The limma program of *R* software was used to find 23 DEGs associated with the copper apoptosis subtype. The prognosis of 10 genes in the copper apoptosis subtype associated DEGs was assessed using a univariate Cox regression analysis. The network activity of 23 DEGS was investigated (see Figure 2(c)). Based on five copper clusters, the therapy sensitivity of chemotherapy was evaluated (see Figure 2(d)), with significantly different IC50 among five cuprotosisClusters (*p* < 0.001). Thorsson et al. [20] investigated the pan-cancer immune landscape and eventually found the six immune subtypes (C1-C6) considered for determining the immune response patterns and have consequences for future immunotherapy research. In most LUAD patients, the immune subtype C3 was enriched, which is characterized by lower levels of overall CNVs in Figure 2(e). For a more detailed description, we execute 23 new DEGs as the same as Figure 2(c) for network plot in Figure 2(f).

### 3.3. Molecular Subtype Identification in Distinct Cuprotosis Modification Patterns

**Figure 3:**
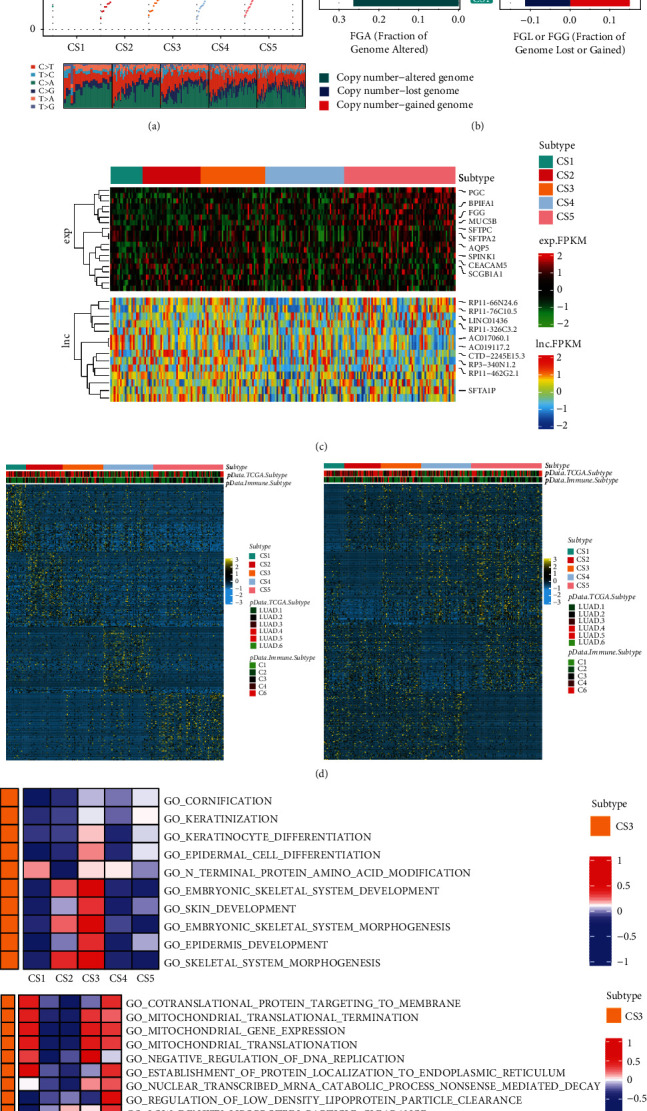
Molecular typing based on the cuprotosis cluster of thyroid cancer. (a) Comparison of TMB and TiTv among four identified subtypes of thyroid cancer in LUAD cohort; (b) bar plot of fraction genome altered among four identified subtypes of thyroid cancer in LUAD cohort; (c) molecular subtypes in distinct cuprotosis clusters. From top to bottom: mRNA expression (median normalized expression levels); lncRNA expression (median normalized expression levels); (d) heatmap of subtype-specific upregulated and downregulated biomarkers using limma for 5 identified subtypes in LUAD cohort; (e) GSVA of subtype-specific upregulated pathways (left). GSVA of subtype-specific downregulated pathways in LUAD cohort (right).

In comparison to the other clusters, cuprotosisCluster-mc1 had a higher level of TMB, overall CNVs, and specific lnc- and m-RNA expression profile (see Figures 3(a), 3(b), and 3(c)). The aneuploidy score and overall CNVs were highest in cuprotosisCluster-C2, and low in cuprotosisCluster-C3. For further exploration, different cuprotosisCluster subtypes with potential predictive biomarkers and functional pathways were characterized. Subtype-specific upregulated or downregulated biomarkers were found by starting with differential expression analysis (DEA). The most DEGs sorted by log2Fold are chosen as the biomarkers for each cuprotosisCluster subtype. These biomarkers should pass the R/limma analysis to identify subtype-specific downregulated Figure 3(d) in left and upregulated in right biomarkers.

Similarly, GSEA is run for each subtype based on its corresponding DEA result to identify functional pathways using a gene set background which includes all gene sets derived from GO biological processes (c5. bp.v 7.1. symbols. gmt). Heatmap analysis of subtype-specific downregulated biological pathways is given (see Figure 3(e) top) using limma package for five identified subtypes in LUAD and upregulated pathways (see Figure 3(e) bottom).

### 3.4. Construction of the Cuprotosis Gene Signature and Evaluation of the Molecular and Immune Landscape Was Significantly Associated with cuprotosisScore

**Figure 4:**
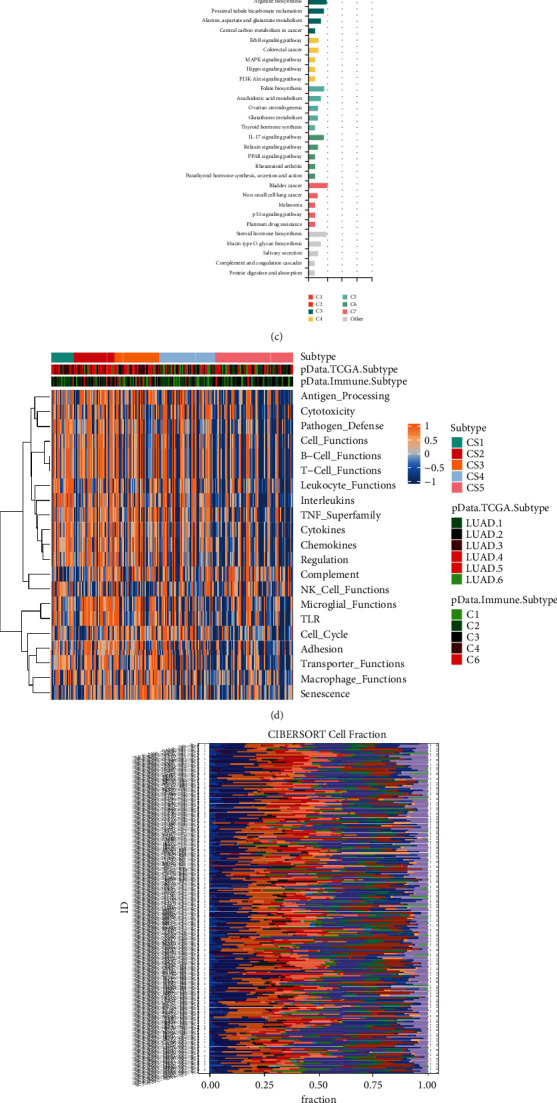
The molecular and immune landscape with distinct cuprotosis modification patterns. (a) Box plot visualizing the cuprotosisSore of five clusters. Boxplot showing the different cuprotosisScore between cuprotosis subtypes. ANOVA test: the asterisks represented the statistical *p* value (^*∗*^*p* < 0.05; ^*∗∗*^*P* < 0.01; ^*∗∗∗*^*P* < 0.001); (b) scatterplot with marginal distributions overlaid on the axes and results from statistical tests in the subtitle for cuprotosisScore and CNV aneuploid score; (c) 192 overlapping DEGs enriched in KEGG pathways; (d) heatmap of enrichment score of gene set of interest for five identified subtypes in LUAD; (e) the distribution of 22 types of immune cells of LUAD using CIBERSORT; (f) heatmap plot showing the 8 different immune-related genes between cuprotosis subtypes.

The immunological properties of various cuprotosis modification patterns were next investigated in further detail. 23 genes associated with significant prognoses were extracted for further PCA analysis to establish the copper apoptosis gene signature. From the visualized box plot (see Figure 4(a)), we could find a positive differentiation (*p* < 0.05) between these five CopperClusters. Furthermore, the Student's *t*-test showed a significant difference in cuprotosisScore among cuprotosis clusters. It was shown that CopperScore was not positively correlated with AS (see Figure 4(b)). We used the cuprotosisScore approach to properly assess the cuprotosis alteration pattern in individual LUAD patients. The limma program of *R* software was used to find 23 DEGs associated with the cuprotosis subtype. The activity of KEGG pathway processes was investigated using GO analysis among these different cuprotosis modification patterns. In DEGs and Cox regression, substantially DEGs were notably enriched in pathways linked to non-small cell lung cancer-related terms, such as p53, MAPK, and PI3K-Akt signaling pathway, as depicted in Figure 4(c). Meanwhile, immune-related pathways such as the IL-17 signaling pathway were shown to be overrepresented among the implicated pathways.

Multiple IM antagonists and agonists are studied in clinical oncology since IMs are important for ICT therapy. Understanding their expression in diverse copper apoptosis alteration patterns is required to progress this research. The functions based on the expression of IM genes in the copper apoptosis subtypes were investigated (see Figure 4(d)). Almost all functions were strongly expressed in mc1, especially in immune functions, such as *T* function, B function, APC processing, and macrophage functions. Using the cibersort algorithm, the bar plot of immune cells in LUAD tissues is shown in Figure 4(e). The heatmap of immune-related genes shows higher expression in mc1 than mc2345 cancer samples as shown in Figure 4(f).

## 4. Discussion

Published research studies had reported that cuprotosis genes showed their crucial biological and clinical functions on tumor development, clinical therapeutic resistance, and immune-oncology response via cross-work among the cuprotosis regulators. Currently, the effects of modification patterns on the TIME were explored in some cancer types. [1] In our study, the role of cuprotosis modification in the immune landscape of LUAD was profiled to deepen our knowledge of the immune-oncology response based on LUAD iTIME and provide more potentially effective ICT clinical treatment strategies.

Based on molecular genotyping by genomic profiling [21, 22], the future clinical application for LUAD patients has been improved. In this study, five cuprotosis modification clusters with significantly distinct TIME were identified based on 10 cuprotosis gene regulators, including different drug treatment sensitivity, the differences in aneuploidy, overall somatic copy number variation, and expression level of the immune-related genes and clinical prognosis (OS). In our study, cuprotosiscluster-mc1 showed enrichment pathways related to full immune activation and relatively high T-cell function, suggesting high tumor growth rates in mc1. Accordingly, it was not shown that C3 exhibited deactivated immunity but a poor survival prognosis. For the clinical application of LUAD patients, we applied a methodology, known as cuprotosisScore, of individual LUAD patients, to exactly indicate the cuprotosis methylation level. After an integrated analysis, it was revealed that cuprotosisScore can be a potential and independent prognostic factor for LUAD patients. In this study, we verified the clinical value of the cuprotosisScore in the cold immune status (cuprotosiscluster C3) LUAD patients. As is known, the pre-existing CD8+ T cell infiltration and a high TMB drive the response to anti-PD-1/PD-L1 ICT therapy. [23] Thus, combined with our results, the cuprotosisScore may serve as a potential indicator for ICT therapy.

Finally, this investigation discovered a link between cuprotosis alteration, tumor mutation burden, and the immunological landscape of LUAD tumors. Our in-depth analysis of cuprotosis alteration patterns in individual LUAD patients adds to our knowledge of the tumor immunological landscape and paves the way for novel and better immunotherapeutic methods for LUAD patients. With consideration of the lack of clinical cohorts to verify our current results, further validation based on large-cohort prospective clinical trials is needed in future exploration.

## Data Availability

All data used in this work can be acquired from the GDC portal (https://portal gdc cancer gov/), Broad GDAC Firehose (https://gdac. broadinstitute org/), and the website (https://gdc. cancer. gov/about-data/publications/panimmune).
